# Skeletal muscle pathology in Huntington's disease

**DOI:** 10.3389/fphys.2014.00380

**Published:** 2014-10-06

**Authors:** Daniel Zielonka, Izabela Piotrowska, Jerzy T. Marcinkowski, Michal Mielcarek

**Affiliations:** ^1^Department of Social Medicine, Poznan University of Medical SciencesPoznan, Poland; ^2^MRC National Institute for Medical ResearchLondon, UK; ^3^Department of Medical and Molecular Genetics, King's College LondonLondon, UK

**Keywords:** Huntington's disease, peripheral pathology, skeletal muscle atrophy, disease modifying treatment

## Abstract

Huntington's disease (HD) is a hereditary neurodegenerative disorder caused by the expansion of a polyglutamine stretch within the huntingtin protein (HTT). The neurological symptoms, that involve motor, cognitive and psychiatric disturbances, are caused by neurodegeneration that is particularly widespread in the basal ganglia and cereberal cortex. HTT is ubiquitously expressed and in recent years it has become apparent that HD patients experience a wide array of peripheral organ dysfunction including severe metabolic phenotype, weight loss, HD-related cardiomyopathy and skeletal muscle wasting. Although skeletal muscles pathology became a hallmark of HD, the mechanisms underlying muscular atrophy in this disorder are unknown. Skeletal muscles account for approximately 40% of body mass and are highly adaptive to physiological and pathological conditions that may result in muscle hypertrophy (due to increased mechanical load) or atrophy (inactivity, chronic disease states). The atrophy is caused by degeneration of myofibers and their replacement by fibrotic tissue is the major pathological feature in many genetic muscle disorders. Under normal physiological conditions the muscle function is orchestrated by a network of intrinsic hypertrophic and atrophic signals linked to the functional properties of the motor units that are likely to be imbalanced in HD. In this article, we highlight the emerging field of research with particular focus on the recent studies of the skeletal muscle pathology and the identification of new disease-modifying treatments.

## Introduction

Huntington's disease (HD) is neurodegenerative disorder caused by the expansion of polyglutamine stretch within the huntingtin protein (HTT) (Gusella et al., 1993; Vonsattel and DiFiglia, 1998; Novak and Tabrizi, 2010). The disease is caused by the expansion of a CAG repeat to over 35 CAG repeats in exon1 of the huntingtin (*HTT)* gene which are normally observed in healthy objects. Neurodegeneration, particularly widespread in the striatal nuclei, basal ganglia and cereberal cortex, is a source of neurological symptoms that involve motor, cognitive and psychiatric disturbances (Novak and Tabrizi, 2010). This leads to a wide-range of clinical features including personality changes, motor impairment, dementia and weight loss that are likely to progress over the course of 15–20 years to death (HDRG, 1993; Walker, 2007). In mammals HTT is expressed in many tissues and organs (Hoogeveen et al., 1993; Strong et al., 1993; Trottier et al., 1995). HTT has been identified to be involved in many critical cellular processes like transcription, protein trafficking and vesicle transport (Li and Li, 2004). In mice HTT deletion is embryonically lethal, leading to defects in all germ layers (Zeitlin et al., 1995). It has been established that HTT is affecting organelles and functional systems that are essential for all type cells i.e., mitochondria, ubuquitin-proteasome system and this phenomena is not tissue specific (Li and Li, 2004; Sassone et al., 2009; van der Burg et al., 2009; Zielonka et al., 2014). A recent study of the heart function in two HD mouse models identified pronounced contractile heart dysfunction, which might be a part of dilatated cardiomyopathy (DCM). This was accompanied by the re-expression of fetal genes, apoptotic cardiomyocyte loss and a moderate degree of interstitial fibrosis (Mielcarek et al., 2014). Therefore it is likely that the peripheral pathology of HD, such as weight loss and severe skeletal muscle atrophy, might have a significant input to the disease progression.

## Skeletal muscle pathology in HD patients

A case-study report showed that a semi-professional marathon runner (43 CAGs) developed signs of a slowly progressing myopathy with elevated creatine kinase levels many years before first signs of chorea were detected. Muscle biopsy revealed a mild myopathy with mitochondrial pathology including a complex IV deficiency (Kosinski et al., 2007). The isometric muscle strength of 6 lower limb muscle groups was measured in 20 people with HD and matched healthy controls. HD patients had reduced muscle strength by 50% on average in comparison to healthy matched controls (Busse et al., 2008). Several studies have reported defects in the mitochondrial function of the central nervous system and skeletal muscles in HD patients (Reddy, 2014). For example, the non-invasive method of 31P-MRS (31P- Magnetic Resonance Spectroscopy) showed a reduced phosphocreatine to inorganic phosphate ratio in the symptomatic HD patients at rest. Muscle ATP/phosphocreatine and inorganic phosphate levels were significantly reduced in both symptomatic and presymptomatic HD subjects (Lodi et al., 2000). During recovery from exercise, the maximum rate of mitochondrial ATP production was reduced by 44% in the symptomatic HD patients and by 35% in the presymptomatic HD carriers. HD subjects showed also a deficit in the mitochondrial oxidative metabolism and that might support a role for mitochondrial dysfunction as a key factor involved in the HD-related muscle pathogenesis (Lodi et al., 2000; Saft et al., 2005). In addition, the total exercise capacity was normal in HD subjects but notably the presymptomatic HD patients had a lower anaerobic threshold and increased level of plasma lactate (Ciammola et al., 2011). *In vitro* muscle cell cultures revealed that HD cells produced more lactate and that might be indicative of a higher glycolysis level (Ciammola et al., 2011). Furthermore, muscle cultures showed cellular abnormalities including mitochondrial membrane potential, cytochrome c release, increased CASPASE-3, −8, and −9 levels and defective cell differentiation, likely due to the formation of HTT inclusions in differentiated myotubes (Ciammola et al., 2006). Finally, electron microscopy showed striking mitochondrial defects like abnormally elongated and swollen mitochondria with derangement of cristae and vacuoles (Ciammola et al., 2011).

## On the way to understand muscle pathology in HD—an animal model

Recent years of research on HD pathogenesis resulted in a generation of many HD transgenic mouse models including mHTT N-terminal fragments and full-length murine or human mHTT (Crook and Housman, 2011; Lee et al., 2013; Rattray et al., 2013). R6/2 mice are transgenic for a mutated N-terminal exon 1 HTT fragment and are the most frequently used in pre-clinical settings. Behaviorally, R6/2 animals at first display a spatial learning deficit at 3–4 weeks of age (Lione et al., 1999). Attention learning deficits and abnormal performance in motor tests (swimming and high speed rotarod) appear at 5–6 weeks of age (Carter et al., 1999; Lione et al., 1999; Murphy et al., 2000), followed by development of a resting tremor, gait disturbances and visual learning deficits at 8–9 weeks of age (Carter et al., 1999; Murphy et al., 2000). There is support that mHTT may have detrimental effects in the skeletal muscles of R6/2 mice due to poly(Q) aggregate accumulation (Sathasivam et al., 1999; Moffitt et al., 2009) and formation of these inclusions in myoblasts and myotubes have been confirmed *in vitro* (Orth et al., 2003). Alternatively, the toxicity of triplet repeat-containing RNA and/or patially mis-spliced huntingtin gene (*Htt*) could be considered as an additional mechanism of HD pathology (Sathasivam et al., 2013).

Transcriptional deregulation is a typical feature of HD pathology in the brain (Luthi-Carter et al., 2002). A similar transcriptional profile in skeletal muscles (quadriceps) from R6/2 mice, *Hdh*Q150 homozygous knock-in mice and HD patients has been identified and that was consistent with a transition from fast-twitch to slow-twitch muscle fiber types (Luthi-Carter et al., 2002). On the other hand, based on immunohistochemistry both type I and II muscles were atrophic. Although atrophy occurred in both type fibers, there was more type I fibers in the R6/2 skeletal muscles. Hence, there was a conversion of type II fibers to type I during the process of muscle atrophy (Ribchester et al., 2004). However, these findings in pre-clinical settings are inconsistent with an increased glycolysis observed in human patients (Ciammola et al., 2011). Metabolic adaptations similar to those induced by diabetes or fasting are also present in HD mouse models but neither metabolic disorder could explain the full phenotype of HD muscle (Strand et al., 2005). Consequently, at the ultrastructural level, the sciatic nerve displayed abnormalities in large myelinated fibers in the presymptomatic R6/2 mice. A significant decrease in the axoplasm diameter of myelinated neurons and increased number of degenerating myelinated fibers were observed; although myelin thickness and unmyelinated fiber diameter were not affected (Wade et al., 2008). The synaptic transmission at the neuromuscular junction has also been studied in the R6/1 mouse model of HD. The morphological data suggest that the innervation pattern of the neuromuscular junctions in R6/1 muscles were normal in early symptomatic animals. However, the size and frequency of miniature endplate potentials were not changed in the R6/1 mice, while the amplitude of evoked endplate potentials increased. Consistent with a pre-synaptic increase of release probability, synaptic depression under high-frequency was higher in R6/1 mice. No changes were detected in size and dynamics of the recycling synaptic vesicle pool (Rozas et al., 2011). In contrast, it has been shown that skeletal muscles of R6/2 mice developed age-dependent denervation-like abnormalities, including reduced endplate area, supersensitivity to acetylcholine, decreased sensitivity to mu-conotoxin and anode-break action potentials (Ribchester et al., 2004). Moreover, the miniature endplate potential (mEPP) amplitude was notably increased while mEPP frequency was significantly reduced in R6/2 mice. Severely affected R6/2 mice developed a progressive increase in a number of motor endplates that fail to respond to nerve stimulation but there was no constitutive sprouting of motor neurons, even in severely atrophic muscles. In fact there was no age-dependent loss of regenerative capacity of motor neurons in R6/2 mice (Ribchester et al., 2004). Another group has studied the membrane properties of skeletal muscles that control contraction in the same HD mouse model. Adult skeletal muscle from R6/2 mice showed that the action potentials in diseased muscles were more easily triggered and prolonged than in wild type littermates. Furthermore, the expression of the muscle chloride channel (ClC-1) and *Kcnj2* (Kir2.1 potassium channel) transcripts were significantly reduced and defects in mRNA processing were detected (Waters et al., 2013).

To better understand a mechanism underlying muscle wasting in the R6/2 mouse model, key pathways governing protein metabolism, apoptosis and autophagy were examined. R6/2 mice exhibited increased adiposity and elevated energy expenditure without altered food intake. A total protein synthesis was unexpectedly increased in the gastrocnemius muscle by 19%, which was associated with over-activation of rapamycin mTOR signaling (She et al., 2011). The transcript levels of androgens, like muscle ring finger-1 and atrophy F-box, were markedly attenuated during fasting and re-feeding. Additionally, the mRNA level of several caspase genes involved in both extrinsic and intrinsic apoptotic pathways, like CASPASE-3/7, −8, and −9, were elevated (She et al., 2011). Indeed, the CASPASE-6 up-regulation might be due to enhanced activity of the p53 in the muscles obtained from HD patients and from two different HD mouse models. It has been also shown that CASPASE-6 may target (cleave) laminin A (Ehrnhoefer et al., 2014). It was suggested that this phenomenon might be mitigated by a small molecule pifithrin-alpha, an inhibitor of p53 transcriptional activity (Ehrnhoefer et al., 2014).

Since mitochondrial dysfunction might play a crucial role in HD pathology (Quintanilla and Johnson, 2009), the role of PPAR γ coactivator 1α (PGC-1α) has been carefully assessed (Lin et al., 2005). Reduced levels of PGC-1α and its target genes in skeletal muscles of HD transgenic mice and HD subjects have been found. Treatment with guanidinopropionic acid (GPA) led to an increased expression level of AMPK, PGC-1α target genes and the genes characteristic for oxidative phosphorylation, electron transport chain and mitochondrial biogenesis. Oxygen consumption in response to GPA treatment was significantly reduced in myoblasts from HD patients (Chaturvedi et al., 2009). On the other hand, knockdown of mutant *HTT* resulted in increased PGC-1α expression in HD myoblast, while PGC-1α rescue led to increased expression of markers for oxidative muscle fibers and reversal of blunted response for GPA in HD mice (Chaturvedi et al., 2009). These findings showed that impaired function of PGC-1α plays a critical role in the skeletal muscles dysfunction in HD. Also, possible pharmacologic intervention with a small molecule could enhance PGC-1α function may exert therapeutic benefits (Chaturvedi et al., 2009). In addition, atrophic fibers of R6/2 mice showed increased fuchsinophilic aggregates and reduced cytochrome *c* oxidase by 15%. Complex I–dependent respiration of HD mitochondria showed more sensitivity to inhibition by Ca^2+^ than in wild-type mitochondria (Gizatullina et al., 2006). A summary of morphological and molecular characteristics of skeletal muscles in pre-clinical and clinical settings has been presented in Table 1.

**Table 1:** **Summary of defects observed in muscle in the pre-clinical and clinical HD settings**.

	**Human**	**Mouse models**
Reduced muscle strength	✓	✓
Muscle atrophy	Unknown	✓
Mitochondrial dysfunction	✓	✓
Inclusions formation	✓	✓
Transcriptional deregulation	✓	✓
Fast to slow twitch	unknown	✓
Increased of adiposity and protein synthesis	unknown	✓
Neuro-muscular junctions abnormalities	unknown	✓
Increased caspase activity	✓	✓

## Can we delay HD progression by modulating muscle function?

As it has been mentioned in the previous paragraph, enhancing PGC-1α activity might be a good strategy to improve skeletal muscles function in HD. Indeed, pharmacologic treatment with the pan-PPAR agonist bezafibrate restored the PGC-1α, PPARs and downstream genes to wild type levels. It also prevented conversion of type I oxidative to type II glycolytic muscle fibers as well as increased muscle mitochondria numbers. Finally, bezafibrate rescued lipid accumulation and apparent vacuolization of brown adipose tissue in the HD mice (Johri et al., 2012).

The other strategy to improve muscle function in HD is based on the heat shock machinery modulation that could suppress mHTT aggregation (Labbadia and Morimoto, 2013). The R6/2 mice expressing an active heat shock transcription factor 1 (HSF1) isoform had reduced polyglutamine inclusion formation and improved body weight. Unexpectedly, the lifespan of R6/2:HSF1Tg mice were significantly improved despite the fact that active HSF1 was not expressed in the brain. These results indicated that active HSF1 has a strong inhibitory effect on polyglutamine aggregates formation *in vivo* (Fujimoto et al., 2005).

Recent studies also showed that HDAC4 function in the cytoplasm (Mielcarek et al., 2013a) and its reduction, delayed cytoplasmic aggregate formation and rescued neuronal and cortico-striatal synaptic function in HD mouse models. This was accompanied by an improvement in motor co-ordination, neurological phenotypes and increased lifespan (Mielcarek et al., 2013b). Given that HDAC4 has well-established functions in skeletal muscle, muscle atrophy is a major symptom of HD and that HDAC4 has been linked to disease progression in an ALS mouse model, it is likely that genetic reduction of HDAC4 in skeletal muscle was a contributing factor to the improved HD phenotypes (Bruneteau et al., 2013).

## Summary

**Figure 1:**
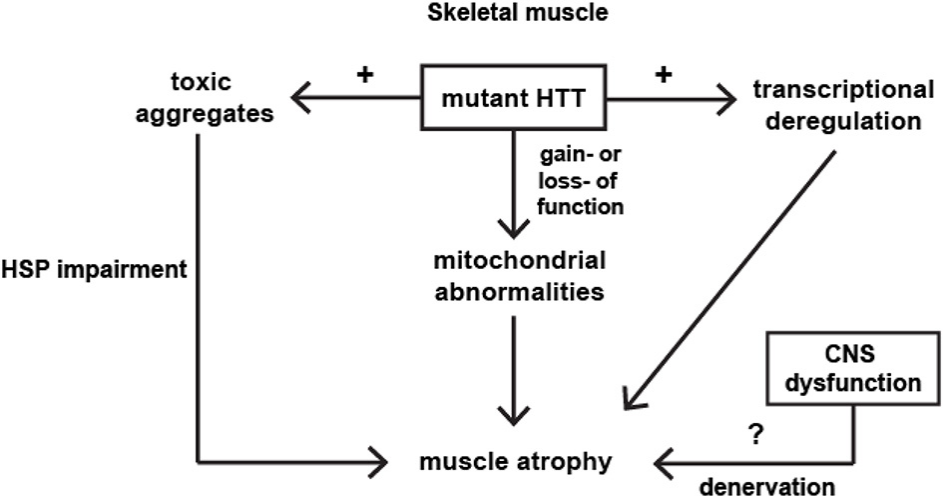
**Summary of pathological events identified in the skeletal muscles in HD**.

HD is a complex disease that has a peripheral component to its pathophysiology. Transcriptional changes in the HD skeletal muscles were comparable to those observed in the different brain regions and skeletal muscle wasting/atrophy is likely to be an important portion of HD pathogenesis. Some of the molecular and physiological changes in HD muscles can be detected, even in the pre-symptomatic HD individuals. On the molecular level, mitochondrial dysfunctions, PPAR alpha signaling and HSF1 activation were identified as major players in the muscle HD-related pathology. The major pathological pathways identified in skeletal muscles have been summarized in Figure 1. However, many aspects of HD neuromuscular transmission and muscle physiology remain unanswered and need to be studied more extensively. The proof of concept studies clearly showed that by improving muscle function in HD mouse models, the progression of disease onset could be delayed and the lifespan extended. Therefore this makes skeletal muscles an attractive target for future therapies. Two key signaling pathways, i.e., Insulin like growth Factor IGF and GDF-8/myostatin, have emerged in recent years to be potent regulators of skeletal muscle size. Moreover, several studies emphasized a role of hyperacetylation in muscle wasting. Therefore there is a need for more pre-clinical and clinical studies that will unravel the mechanism of HD skeletal muscles pathology, leading to potential therapies in HD. Further work is necessary in order to fully appreciate the complexity of the pathways that are affected during HD progression. Indeed, emerging evidence has clearly indicated that peripheral tissues are as much affected by the expression of the mutant huntingtin as the Central Nervous System. Furthermore, the possibility to test the effect of new drugs directly on human peripheral tissues is a new and exciting research area.

### Conflict of interest statement

Conflict of Interest Statement: The authors declare that the research was conducted in the absence of any commercial or financial relationships that could be construed as a potential conflict of interest.
